# Cardiovascular Imaging in Pregnancy: Valvulopathy, Hypertrophic Cardiomyopathy, and Aortopathy

**DOI:** 10.3389/fcvm.2022.834738

**Published:** 2022-08-03

**Authors:** Haneen Ismail, Andrew J. Bradley, Jannet F. Lewis

**Affiliations:** Division of Cardiology, Department of Medicine, George Washington University School of Medicine and Health Sciences, Washington, DC, United States

**Keywords:** cardiac MRI (CMR), cardiac CT, pregnancy, hypertrophic cardiomyopathy, valvulopathy, aortopathy, echocardiography, peripartum

## Abstract

Pregnancy is associated with profound hemodynamic changes that are particularly impactful in patients with underlying cardiovascular disease. Management of pregnant women with cardiovascular disease requires careful evaluation that considers the well-being of both the woman and the developing fetus. Clinical assessment begins before pregnancy and continues throughout gestation into the post-partum period and is supplemented by cardiac imaging. This review discusses the role of imaging, specifically echocardiography, cardiac MRI, and cardiac CT, in pregnant women with valvular diseases, hypertrophic cardiomyopathy, and aortic pathology.

## Introduction

Pregnancy is associated with profound hemodynamic changes that significantly impact the cardiovascular system. These changes are particularly impactful in women with underlying heart disease. Management of pregnant women with cardiovascular abnormalities requires consideration of the well-being of both the woman and the developing fetus. Clinical evaluation, starting before and then continuing throughout pregnancy, is the foundation of this management with imaging playing a key role in patient risk stratification and monitoring. This review discusses the role of imaging for pregnant women with valvular diseases, hypertrophic cardiomyopathy, and aortic pathology. We will begin with a brief overview of general patient assessment and discussion of the value added by different imaging modalities.

## A Basic Approach to Patient Management and Imaging Selection

Preconception evaluation of possible maternal and fetal risks during pregnancy is important to help tailor plans for maternal and fetal monitoring and identify the level of care needed during labor and in the post-delivery period. Maternal cardiac risk can be assessed using three main models including the World Health Organization (WHO) risk model, the Cardiac Disease in Pregnancy (CARPREG) risk score and the Zwangerschap bij Aangeboren HARtAfwijkingen I (ZAHARA) risk score (1–3). The three models share common features, such as incorporating lesion-specific predictors, but differ in a few key areas (1, 2). The World Health Organization (WHO) classification categorizes women into four different classes based on specific congenital and acquired heart disease, with WHO Class IV being the highest risk for mortality where pregnancy is contraindicated. The CARPREG risk score emphasizes left-sided cardiac obstruction in stratifying pregnant women for adverse cardiac and fetal outcomes. The ZAHARA risk score includes all moderate and severe valvular lesions and mechanical valves in the risk prediction model (1). Assessment of risk is of course based on careful clinical assessment, but necessarily includes non-invasive imaging.

The choice of imaging modality first depends on the pathology or pathophysiology to be evaluated. Echocardiography remains the mainstay for assessment of valvular disease and non-ischemic cardiomyopathies but is less comprehensive for assessment of aortopathy (4). Advanced imaging modalities including contrast-enhanced computed tomography (CT) and magnetic resonance imaging (MRI) provide important corroborating and incremental information, particularly in patients with suspected or known aortopathy. MRI and CT provide primarily anatomic information, although cardiac MRI can provide valuable physiologic information as well. Table 1 summarizes the advantages and disadvantages of imaging modalities.

**TABLE 1:** The advantages and the disadvantages of each imaging modality in pregnancy.

Modality	Echocardiography	Contrast-enhanced CT	MRI without contrast
Pathology evaluated	• Valve stenosis • Valvular regurgitation • Proximal aortopathy • Ventricular function	• Aortopathy (full visualization)	• Ventricular function and specific cardiomyopathies • Aortopathy (full visualization) • Valve dysfunction
Advantages	• Easily available • No ionizing radiation • No gadolinium-based contrast	• Excellent resolution • Complete visualization of aorta	• Excellent resolution • No ionizing radiation • Complete visualization of aorta
Disadvantages	• Acoustic windows may be compromised during pregnancy • Limited visualization of thoracic aorta	• Ionizing radiation • Intravenous contrast for assessment of aorta	• Relatively long scan times • Image quality adversely affected by arrhythmias
			

**FIGURE 1:**
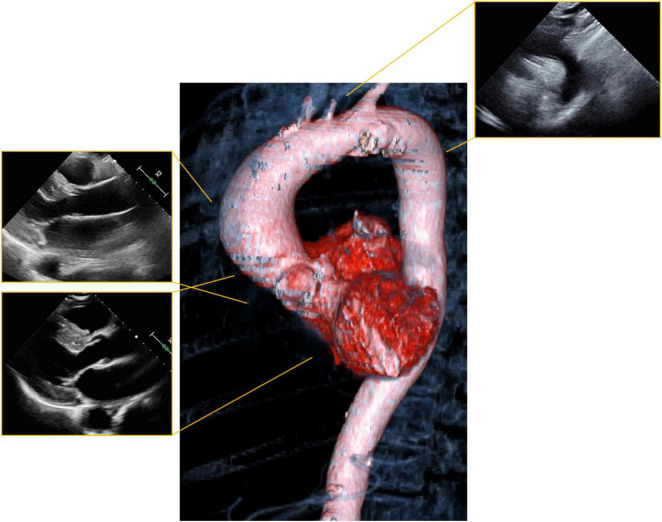
Visualization of the aorta with echocardiography. Standard parasternal and suprasternal notch views (periphery) leave a significant portion of the aorta unvisualized. Computer tomography (CT) or magnetic resonance imaging (MRI) can visualize the aorta in three dimensions (center) and allows for comprehensive evaluation of anatomy.

The choice of imaging modality is therefore based on the reliability and accuracy of the modality for evaluation of the pathology in question, as well as safety for the mother and fetus. Echocardiography has essentially no safety concerns for the mother or fetus during pregnancy and remains the fundamental imaging modality for assessment of myocardial and valvular disease. However, imaging may be limited due to patient positioning and the gravid uterus (4). In particular, echocardiographic visualization of the aorta is often limited to the aortic root and proximal ascending aorta (Figure 1). Although echocardiography offers the greatest safety from a procedural standpoint, this must be weighed against the risk of missed or inadequately-characterized aortic pathology. We briefly review the existing data regarding use of CT and MRI in pregnancy.

Clinical MRI at 1.5 T has not been proven to confer any significant risk on the fetus. Studies performed at 3 T are considered safe at up to 30 min of scan time (5). A non-contrast cardiac MRI can provide a significant amount of clinically important information: accurate evaluation of cardiac chamber sizes, quantification of flows and regurgitant volumes, and delineation of aortic anatomy. The risk-benefit ratio for gadolinium-based contrast agents, on the other hand, is less clearly defined. One study found that gadolinium exposure during gestation was associated with an increased risk of stillbirth, neonatal death, and certain rheumatologic, dermatologic, and inflammatory conditions, therefore, guidelines recommend avoiding routine administration of GBCAs to pregnant patients (5, 6).

Cardiac CT uses ECG-gating to obtain high quality images of the heart and surrounding vasculature. Prospective gating provides a “freeze frame” evaluation of the heart and dramatically reduces radiation doses - approximately 1 milligray (mGy) to the fetus. Retrospective gating allows for evaluation of cardiac wall and valve motion but at approximately 3 mGy to the fetus (7). In general, the risk of malignancy due to radiation depends both on dose and life expectancy after radiation. Thus, for a given dose of radiation, a young woman of child-bearing age is at higher risk of eventually developing malignancy from medical radiation than an older, post-menopausal woman. When radiologic studies are necessary, the goal is to follow the ALARA (as low as reasonably achievable) principle, minimizing radiation dose as much as possible while still obtaining diagnostic images. The risks of ionizing radiation to a fetus vary with gestational age and dose. In general, doses of < 50 mGy pose insignificant risk to the fetus (8).

For conditions discussed in this review, CT scanning must be performed with contrast agents to provide diagnostically meaningful images. Iodinated contrast media can cross the placenta but there have been no proven adverse effects on fetal development. Therefore, contrast-enhanced CT scanning should not be withheld from a patient due to pregnancy (7). Rather, risk-benefit discussions should be held, comparing the theoretical risks to the fetus against the risks to both the patient and fetus if a crucial diagnosis is missed.

With an understanding of the value added by different imaging modalities, we will next discuss the management of specific conditions.

## Aortic Valve Disorders

### Aortic Valve Stenosis

Cardiac output and stroke volume increase during pregnancy and reach their peak during the second to third trimester. Output and stroke volume may be increased up to 45% in a normal pregnancy and an estimated additional 15% higher in twin pregnancy (9, 10). These hemodynamic changes lead to increases in valvular gradients (11). Women with severe aortic stenosis (AS) are more likely to develop heart failure and adverse fetal outcomes including preterm birth and low birth weight (12). As hemodynamics change during pregnancy, so too does the risk of heart failure, particularly during the period from the second trimester through the first 24–72 h postpartum (13).

Congenital aortic stenosis can be seen from bicuspid aortic valve, subvalvular, and supravalvular stenosis, but overall is well-tolerated during pregnancy (14). Additionally, bicuspid aortic valve and associated aortopathy or coarctation of the aorta may be seen together in women with Turner’s syndrome (15).

#### Preconception Planning

The diagnosis of severe AS is made by echocardiography using standard methods to determine aortic valve gradients. Symptomatic severe aortic stenosis is a particularly high-risk condition (12). For example, the estimated risk of primary cardiac events in a woman with severe, symptomatic aortic stenosis using the CARPREG II score is 10%. In circumstances where pregnancy is deemed high risk for maternal and or fetal mortality, a collaborative approach with obstetric and cardiovascular specialists should include discussion of non-surgical or surgical intervention (16). Contraceptive approaches should also be discussed for high-risk women of child-bearing age (17).

Asymptomatic women with severe aortic stenosis are also not without significant risk. Although data are limited, evaluation with exercise echocardiography or cardiopulmonary exercise testing is recommended to evaluate the hemodynamic response to increased demand. Women with bicuspid aortic valve, without or without significant aortic stenosis, should have careful assessment of the thoracic aorta before pregnancy (16).

#### During Pregnancy

Given the dynamic physiological changes throughout pregnancy, clinical monitoring and serial echocardiography are appropriate. Clinical evaluation should assess for development of AS symptoms such as fatigue, shortness of breath, syncope, presyncope, or other heart failure symptoms (18).

Echocardiography once every trimester is generally adequate and most importantly, is recommended at the time of peak hemodynamic load estimated to be at about 32 weeks gestation (13). Congenital bicuspid aortic valve is associated with coarctation of the aorta in about 10% of patients and aortopathy is common. Therefore, the aorta should be carefully examined by echocardiography as well. Transesophageal echocardiography is safe in pregnancy but the aspiration risk is somewhat elevated compared to normal due to an increase in intra-abdominal pressure (19).

Cardiac CT is usually not necessary for diagnosis or planning management of AS but can be considered in women with bicuspid aortic valve when evaluation for aortopathy is indicated. A low radiation CT with a fetal dose of 0.01–0.66 mGy can be used to evaluate for aortic diameters before and during pregnancy (20). MRI generally does not add significantly to diagnosis or management of AS during pregnancy, and usually is not required or advised unless other diagnostic imaging are insufficient. Cardiac catheterization is rarely needed for diagnosis, but necessary if intervention must be performed for severe symptomatic AS in pregnancy. Fetal exposure to radiation from cardiac catheterization is low – the abdomen, if unshielded, receives 1.5 mGy on average with less than 20% reaching the fetus (21).

#### Management

Increased blood volume in pregnancy causes elevated gradients across the stenosed aortic valve and may result in development of decompensated heart failure (HF). Management includes use of loop diuretics for relief of vascular congestion, but they should be used with caution to avoid hypoperfusion of the placenta. According to the ACC/AHA guidelines, women with valvular disease undergoing uncomplicated vaginal delivery or Cesarean section do not require prophylactic antibiotics (16).

In severely symptomatic women with aortic stenosis who do not respond to medical management, aortic balloon valvuloplasty or transcatheter valve replacement can be considered in patients with favorable anatomy (11, 22). If aortic valvuloplasty is determined to be the best management approach, the best timing is after organogenesis is complete which is after the 4*^th^* month of pregnancy (21). There are limited data on these interventional approaches, but case reports suggest their feasibility. Berry et al. reported a case of a 33 year-old woman at 22 weeks gestation with a 21-mm Carpentier Edwards Magna valve bioprosthetic aortic valve who developed progressive symptomatic AS during her pregnancy and a mean aortic valve gradient of 61 mmHg. She successfully underwent a valve-in-valve transcutaneous aortic valve replacement using a 20-mm Edwards Sapien 3 valve with significant improvement in clinical symptoms and reduction of the mean aortic valve gradient to 23 mmHg. At 37 weeks gestation, she delivered a healthy baby by Cesarean section (11). Orwat et al. reported a successful aortic balloon valvulotomy in a woman with severe AS at 20 weeks of gestation leading to symptomatic systolic heart failure. Pregnancy was subsequently uneventful though she required aortic valve replacement 1 month after delivery for recurrent symptoms (12). Surgical aortic valve replacement during pregnancy is exceptionally rare because of the reported risk of fetal mortality associated with cardiopulmonary bypass but has also been reported with success (23).

#### Labor and Delivery

The hemodynamic changes of pregnancy peak during labor and delivery with an increase in stroke volume and cardiac output up to 80% immediately following delivery in response to pain, bleeding, uterine contraction, and anxiety (10, 24). It is estimated that uterine contraction leads to approximately 300–500 ml of placental blood autotransfusion to the systemic circulation which contributes to the increase in both systolic and diastolic blood pressure (10).

In general, vaginal delivery is preferred and Cesarean delivery is not generally recommended unless there is severe aortic stenosis (21, 24). Compared to women with moderate valve disease, those with severe AS have a higher rate of Cesarean section (75.0 vs. 48.3%). Lower birth weight infants were more common in severe AS (35 vs. 6%), believed to be related to the hemodynamic compromise in severe AS that leads to decreased utero-placental blood flow. These infants usually have a lower Apgar score (< 7) (12).

#### Post-Partum Period

In patients with severe AS or symptomatic moderate AS, a 31% increase in complications has been reported in the early post-partum period (i.e., within 24–72 h of delivery) secondary to fluid shifts. These complications include arrhythmia, pulmonary congestion, death, or need for cardiac intervention. There is also an increased chance of deterioration of the diseased aortic valve weeks or months after delivery (25).

### Aortic Valve Regurgitation

Pregnant women with aortic regurgitation are at low risk for cardiac complications and generally tolerate pregnancy, likely due to a decrease in afterload that reduces regurgitant volume (24). Nonetheless, data suggest that a dilated left ventricle with depressed function may predict an increased likelihood of adverse events (26). Aortic regurgitation may be secondary to bicuspid aortic valve with associated aortopathy which are discussed elsewhere in this review.

## Mitral Valve Disorders

### Mitral Valve Stenosis

**FIGURE 2:**
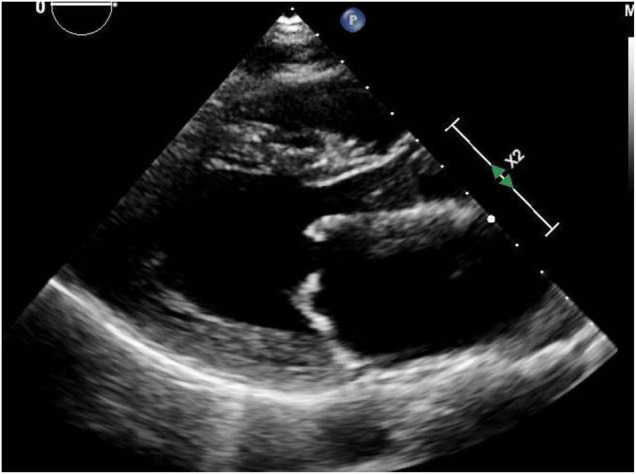
Mitral stenosis: Representative parasternal long axis view during showing mitral stenosis in a woman with rheumatic heart disease. Both leaflets are affected with thickening and also restriction of their movement.

Mitral stenosis most commonly occurs because of rheumatic heart disease, uncommon in developed countries but an important cause in developing nations and in major cities with significant immigrant populations (Figure 2). Congenital mitral stenosis due to parachute mitral valve is rare, and uncommonly seen in adult patients without prior repair. Heart failure symptoms related to mitral stenosis may appear when the mitral valve orifice area is reduced to < 2 cm^2^ (27). The increased stroke volume and cardiac output of pregnancy may unmask previously asymptomatic mitral valve disease (24, 28). The increasing transmitral valve gradient during pregnancy may lead to pulmonary vascular congestion and pulmonary hypertension. Moreover, the increased heart rate associated with pregnancy limits diastolic filling which further worsens pulmonary edema (29).

#### Preconception Planning

A multidisciplinary team consisting of an obstetrician (preferably a high-risk specialist), cardiologist, and anesthesiologist should be involved in the initial evaluation of women with mitral valve stenosis and should follow her closely throughout pregnancy and the post-partum period. The clinical evaluation should include an echocardiogram ideally performed at 6 to 12 months prior to pregnancy. Exercise stress echocardiography may provide additional hemodynamic information in patients without significant symptoms or questionable severity of mitral stenosis (16, 21).

Women should be counseled on the maternal and fetal adverse outcomes associated with mitral stenosis. Left heart obstruction that results from severe mitral stenosis results in uteroplacental insufficiency which consequently leads to increased fetal morbidity and mortality including intrauterine growth retardation (IUGR), low birth weight, preterm delivery and fetal death (30).

The adverse effects in women with mitral stenosis are related to the severity of mitral stenosis and the patient’s NYHA class (27). Increased cardiovascular demand during pregnancy is very poorly tolerated in women with severe mitral stenosis and may result in rapid deterioration of NYHA class (31). More severe stenosis, NYHA III-IV symptoms, LVEF < 40%, and prior cardiac all events are all predictors of adverse cardiac events during pregnancy (28). According to The American College of Cardiology/American Heart Association (ACC/AHA) guidelines, pregnant women who have symptomatic moderate to severe mitral stenosis defined as mitral valve area (MVA) ≤ 1.5 cm^2^ or mean gradient ≥ 5 mmHg are advised to undergo percutaneous balloon mitral valvuloplasty prior to planning conception (16). Women with mitral stenosis on anticoagulation present significant issues during pregnancy. Pregnancy is associated with hypercoagulability and therfore increases the risk of thrombosis which persists up to 12 weeks into the postpartum period. Therefore, it is strongly advised that anticoagulation with heparin bridge to warfarin be resumed in the immediate postpartum period after assessment of bleeding risk and it can be generally started at 6–8 h after uncomplicated vaginal delivery and at 24–36 h after a Cesarean section (32, 33).

#### During Pregnancy

Valve gradients in mitral stenosis increase with increased stroke volume and cardiac output. Thus, for any severity of mitral stenosis, the increased cardiac output occurring during pregnancy results in increased gradients and may cause the severity of mitral stenosis to be overestimated. Therefore, mitral valve assessment should include measurement of mitral valve area, preferably using a method that involves measuring the increased stroke volume (28, 34). Echocardiography once every trimester is generally adequate although consideration must be given to changes in clinical status or symptoms (13). The role of MRI and CT is limited. Mitral valve area can also be obtained by planimetry on cardiac MR or CT, but in general these modalities have limited utility for valve assessment in pregnancy.

#### Management

Pregnancy is generally well tolerated in women with mild or moderate mitral valve stenosis. Women with severe MS should decrease their physical activity and are advised to bedrest during the latter stages of pregnancy (35). Heart failure and pulmonary edema tend to appear during the second or third trimester at the peak increase in cardiac output. In addition to bedrest, management of symptomatic patients should always start with conventional medical therapy for mitral stenosis, including diuretics for volume management and beta-blockade to decrease heart rate and improve diastolic filling. Beta blockers that are safe in pregnancy include propranolol and metoprolol as they do not pose significant fetal adverse effects (36) while atenolol is associated with more fetal growth retardation (28). Diuretics should be used with caution to avoid placental hypoperfusion. All women with mitral stenosis, regardless of severity, should be evaluated for atrial fibrillation. Cardioversion of atrial fibrillation is safe in pregnancy if needed to prevent systemic embolization and to improve left ventricular filling (29). Either warfarin < 5 mg daily and/or low molecular weight heparin can be used for anticoagulation (37).

If intervention is deemed necessary in women with refractory symptoms, the second trimester is the preferred timing. In women without significant mitral regurgitation, percutaneous balloon mitral valvuloplasty results in substantial decrease in valve gradient and increased valve area (38). Open commissurotomy has similar success rate to percutaneous balloon valvuloplasty but poses a high, 8 times greater, risk of fetal loss. Mitral valve replacement is usually the last resort in severe cases in which the valve is not amenable to percutaneous intervention due to heavy calcification or if a mural thrombus is present. Surgical mitral valve replacement carries 1.5–5% risk of maternal mortality and 16–33% risk of fetal loss (29).

#### Labor and Delivery

Vaginal delivery under epidural anesthesia is generally recommended unless otherwise obstetrically contraindicated. Epidural anesthesia reduces tachycardia secondary to labor pain and therefore reduces left atrial pressure and the risk of pulmonary edema during labor. For women in NYHA classes III or IV, invasive hemodynamic monitoring during labor and delivery is helpful in guiding fluid and drug therapy (29). The goal is to allow for uterine contraction while minimizing maternal Valsalva maneuver during expulsive effort. The use of epidural boluses in incremental doses allows for supplemental instrumentation in the second stage of labor to shorten this stage, and most importantly, the slow anesthetic onset allows for maternal compensation to prevent profound hypotension (39). In addition, epidural anesthesia provides segmental blockade which preserves the lower extremity muscle tone, decreasing the incidence of deep venous thrombosis (40).

#### Post-Partum Period

There is a sudden increase in preload during and immediately after delivery due both to relief of pressure of the uterus on the venous circulation and to autotransfusion from the placenta to the maternal central circulation. This may lead to severe pulmonary edema in women with mitral stenosis, and consequently the risk of maternal death is highest during labor and the immediate post-partum period. The increased risk may persist for 24–72 h after delivery until stabilization of the maternal hemodynamic shift (29).

### Mitral Valve Regurgitation

**FIGURE 3:**
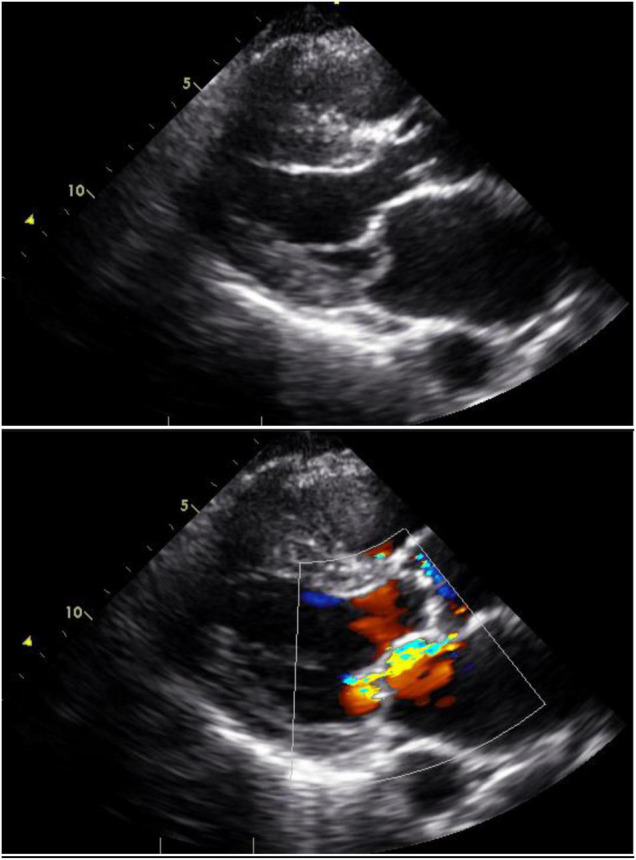
Mitral valve prolapse: Still images with and without color Doppler in a woman with mitral valve prolapse affecting the posterior leaflet. The posterior leaflet bows beyond the mitral annular plane and produces an anteriorly-directed jet of mitral regurgitation.

Mitral regurgitation is most commonly due to mitral valve prolapse (Figure 3). Indeed, primary mitral valve prolapse (MVP) due to myxomatous valve disease is the most common valvular disease in pregnancy (41). MVP is usually suspected or diagnosed with clinical findings of systolic click and mitral regurgitant murmur, but these findings may not be classical in pregnancy because of the volume increases and lower systemic resistance that occur in pregnancy (42, 43). The diagnosis is confirmed and the severity of mitral regurgitation ascertained by echocardiography (Figure 3).

In the absence of other cardiovascular pathology, the majority of pregnant women with MVP and mild or moderate mitral regurgitation have an uneventful pregnancy. Asymptomatic women with mitral valve prolapse and chronic, severe regurgitation usually tolerate pregnancy without significant complication although the increased cardiac volume may be associated with signs and symptoms of volume overload and atrial fibrillation. This can generally be managed with careful diuresis and beta blockers (16, 42, 44). Metoprolol can be safely used in pregnancy but may be associated with fetal bradycardia so should be used judiciously.

Women with mitral regurgitation, depressed LV function, and pulmonary hypertension with pulmonary artery systolic pressure > 50 mm Hg are at high risk for development of heart failure during pregnancy. It is highly recommended that those patients be referred to a specialized valve center for consideration of surgical intervention. Similarly, patients with severe regurgitation and refractory heart failure should also be referred to a specialized valve center (16).

### Right-Sided Valvular Heart Disease

Disease affecting the right-sided cardiac valves occurs much less commonly than left-sided cardiac valves. Involvement of the tricuspid and pulmonic valves in complex congenital heart disease is beyond the scope of this review. Acquired forms of tricuspid valve disease include tricuspid stenosis, a rare complication of rheumatic heart disease, and nearly always occurs with mitral and/or aortic valve involvement. Tricuspid stenosis is usually well tolerated in pregnancy, and the coexistent left sided disease (most commonly mitral stenosis) presents the major issues as discussed above (45). Significant tricuspid regurgitation is also quite uncommon in the absence of congenital heart disease or pulmonary hypertension. The latter poses a particularly high risk to the mother and fetus, and severe pulmonary hypertension should be considered a contraindication to pregnancy. In general, isolated tricuspid regurgitation does not impose clinically significant hemodynamic burden during pregnancy (24).

Isolated pulmonic stenosis is rare and if present is it usually due to branch pulmonary artery stenosis which in turn increases pressure gradient across the pulmonic valve (46). Pulmonic stenosis of mild or even moderate severity may be undetected until adulthood. Severe pulmonic stenosis most commonly occurs in the setting of complex congenital heart disease, typically Tetralogy of Fallot and is beyond the scope of this paper. Pulmonic valve regurgitation without coexistent complex congenital heart disease or pulmonary hypertension is also very uncommon. In general, pulmonic regurgitation with normal right ventricular function is also very well tolerated in pregnancy but is largely influenced by coexistent structural disease (47).

### Mechanical Heart Valves

Mechanical heart valves that necessitate the use of anticoagulation present additional challenges in managing valvular disease in pregnant women. The hypercoagulability associated with pregnancy poses added risk of valve thrombosis. The goal of adequate anticoagulation to prevent valve thrombosis must be balanced with avoiding fetal adverse outcomes. Echocardiography provides important information about prosthetic valve anatomy and function (21). As previously discussed, the increased stroke volume and cardiac output associated with pregnancy can be expected to cause increased transvalvular gradients that should not be misinterpreted as valve dysfunction. Cardiac CT, when performed with retrospective gating, can be used to assess valve motion, though at the cost of radiation (48). Fluoroscopy is another tool for assessment of mechanical valve motion (21). Cardiac MRI has limited use for valve assessment due to magnetic artifact (49).

Warfarin continued throughout pregnancy provides the best protection to the pregnant woman but carries a risk of fetal teratogenicity demonstrated by studies showing that warfarin leads to fetal birth defects if used in the first trimester particularly between weeks 6–12 (50). Current American College of Cardiology/American Heart Association (ACC/AHA) valvular heart disease guidelines recommend the use of warfarin at a daily dose of ≤ 5 mg/day throughout pregnancy, provided adequate anticoagulation is achieved (16). Warfarin at this low dose has been associated with better fetal outcomes and is comparable to low molecular weight heparin (LMWH) (32). Pregnant women who require higher warfarin dosage to maintain a therapeutic anticoagulation are recommended to be switched to low molecular weight heparin given subcutaneously every 12 h, and Anti-Xa levels monitored 4–6 h after a dose (goal range: 0.8–1.2 U/ml) (33). In women on anticoagulation, Cesarean delivery is recommended due to increased risks of neonatal intracranial bleeding during vaginal delivery (21).

In women with mechanical valves, combined hormonal contraception such as pills, patches and vaginal rings carry an increased risk of thrombosis and their use should be discouraged. In contrast, copper intrauterine devices and subcutaneous implants which contain single etonogestrel hormone are safe and are long acting to prevent unintended pregnancy for cardiac patients (51).

### Hypertrophic Cardiomyopathy

#### Preconception Planning

Women with hypertrophic cardiomyopathy should be carefully counseled regarding the risk of HCM in the baby. Nonetheless, pregnancy is generally well-tolerated in patients with hypertrophic cardiomyopathy (52). One study evaluating a cohort of 100 women noted two deaths during pregnancy, both of whom were known to be high risk. One woman had sudden death four days post-partum and was known to have massive left ventricular hypertrophy (i.e., wall thickness 30 mm) with a resting left ventricular outflow gradient of 115 mmHg. The other patient with a particularly malignant family history with eight deaths in young relatives died of ventricular arrhythmia during pregnancy (53). This study illustrates the importance of risk stratification of women with HCM before pregnancy to identify features such as extreme left ventricular hypertrophy, strong family or personal history of sudden cardiac death, syncope, or identified arrhythmias on cardiac monitoring. Echocardiography is recommended in the current guidelines for managing HCM, particularly when new symptoms develop during pregnancy. Patients known to be high risk should also undergo regular echocardiograms while pregnant to assess for significant changes in outflow gradients or left ventricular function (21, 54).

**FIGURE 4:**
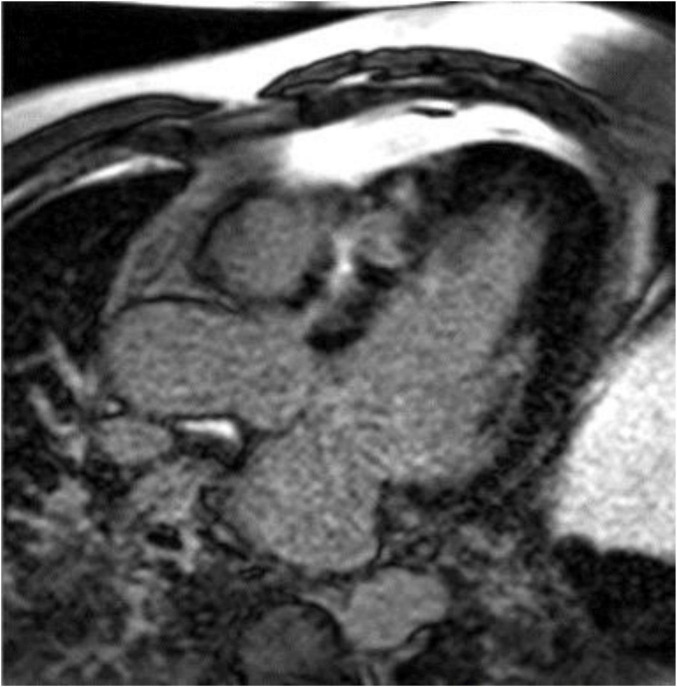
Late gadolinium enhancement (LGE) image from a cardiac magnetic resonance image in the three-chamber view showing significant fibrosis in the anteroseptum of a patient with hypertrophic cardiomyopathy. Image courtesy Arlene Sirajuddin, MD.

Cardiac MR is not specifically recommended by current HCM guidelines for pregnant women or women who may become pregnant. However, CMR does offer advantages over echocardiography, for example by visualizing maximal wall thickness, detection of apical aneurysms, and identification of late gadolinium enhancement (Figure 4) (55). Each of these features has been associated with adverse outcomes in the general HCM population and may plausibly be associated with higher risk during pregnancy. CT has limited recommendations in the guidelines and essentially is considered appropriate for patients who cannot undergo CMR but require imaging beyond echocardiography (56).

#### During Pregnancy, Labor, Delivery, and Post-Partum Period

The increased blood volume occurring in pregnancy is often associated with reduced left ventricular outflow gradient. On the other hand, the increased volume may also result in heart failure in women with severe diastolic dysfunction (57). Diuretics can be used judiciously in women with evidence of vascular congestion (58). Echocardiography may be helpful in management to distinguish symptoms occurring as a normal consequence of pregnancy (for example, dyspnea) and those occurring due to HCM. Thus, evaluation of left ventricular outflow gradient and detection of mitral regurgitation due to systolic anterior motion of the mitral leaflet provide important management adjuncts (59). Most women can undergo uncomplicated vaginal delivery and Cesarean section is reserved for high-risk patients (54).

### Aortopathy

Aortopathy is common in women with bicuspid aortic valve, Marfan’s syndrome, Ehlers–Danlos type IV, Loeys–Dietz syndrome and Turner’s syndrome. The risk of aortic dissection may be significant and related to the degree of aortic dilation (60–62). The risk of dissection appears to be highest during the third trimester, labor and delivery and during the early postpartum period (63). Cardiovascular imaging screening plays a particularly important role in management of these women to assure early identification, need for elective treatment, and avoidance of complications (Figure 1) (64).

#### Preconception Planning

Identification of aortopathy and assessment of severity of aortic dilation is crucial in preconception planning. Bicuspid aortic valve is the most common congenital heart disease (aside from mitral valve prolapse) observed in adults, occurring in 1–2% of the population. BAV can be associated with coarctation of the aorta and aortic dilatation, even with normal valve function. Less common abnormalities such as Marfan’s syndrome, Ehlers–Danlos type IV, Loeys–Dietz syndrome and Turner’s syndrome, are also associated with aortic aneurysm and risk of dissection (63). Echocardiography is recommended for evaluation of the aortic root and proximal ascending aorta. Aortic dilatation involving the distal ascending aorta, arch and descending thoracic is often not well visualized by echocardiography. Therefore, a thorough evaluation of the aorta with CT or MRI in patients with possible aortopathy is strongly recommended for appropriate pre-conception counseling (21). Imaging and prophylactic aortic surgery for an aortic aneurysm can lead to better outcomes and reduce the risk of aortic dissection later in pregnancy. Pregnancy is not recommended in patients with severe dilatation of the aorta in heritable thoracic aortic disease such as Marfan syndrome > 45 mm, bicuspid aortic valve > 50 mm or > 27 mm/m^2^ BSA, or Turner syndrome ASI > 25 mm/m^2^ BSA (21).

#### During Pregnancy

Women with aortic size < 4.0 cm are generally at low risk, but body surface area must be considered (21). Given the morbidity and possible mortality associated with aortic dilation and dissection, radiation concerns should not deter clinicians from proceeding with CT angiography. MRI with or without contrast is also an option based on local availability and expertise.

Pregnant women with aortopathy should undergo serial echocardiography. Echocardiography should be performed monthly in women with significant aortic dilatation (4–4.5 cm for Marfan’s syndrome, 4.5–5.0 cm for bicuspid aortic valve), but is reasonable to be performed every 12 weeks in women with mild aortic dilation. If necessary, because of poor visualization by echocardiography (Figure 1), location of the aortic dilation, or suspected progression, cardiac MRI without contrast can be used for further assessment and follow up (21, 64).

#### Management

Close monitoring, strict blood pressure control, and beta blockers are highly advised throughout pregnancy, particularly among patients with high risk aortopathy (21, 65).

Surgical management is considered when progressive dilatation is observed and in type A aortic dissection and should be planned before fetal viability in the second trimester (62). In cases when progressive dilatation is seen after fetal viability, Cesarean section is recommended followed by aortic reconstructive surgery (63).

#### Labor and Delivery/Post-Partum Period

The goal is to minimize cardiovascular stress, therefore women are advised to continue beta blocker therapy in the peripartum period. Vaginal delivery with expedited second-stage using epidural anesthesia and instrumental delivery is advised in cases with an ascending aorta diameter between 4.0–4.5 cm to prevent abrupt increases in blood pressure and hence reduce risk of dissection. On the other hand, Cesarean delivery is strongly advocated when the aortic diameter exceeds 4.5 cm or in patients with Ehlers–Danlos syndrome type IV due to the high risk of dissection. The increased risk of dissection persists in the postpartum period, and women are recommended to be followed by echocardiography in the immediate post-partum period and 6 months after delivery (21).

## Conclusion

Women with significant valvular heart disease, hypertrophic cardiomyopathy, and aortopathy require careful monitoring that becomes even more important during pregnancy. Careful clinical and appropriate imaging are relevant for risk stratification and surveillance during pregnancy and in the post-partum period.

Echocardiography is the foundation of cardiac imaging during pregnancy. In the conditions described in this review, echocardiography offers safe, readily-available, and diagnostic information to aid in patient management. However, echocardiography may be limited due to acoustic windows and image quality. MRI, including cardiac MRI, performed without gadolinium, can safely offer important diagnostic information, particularly in patients with aortopathy. Cardiac MRI may aid in preconception management of patients with HCM by clarifying anatomy and assisting with risk stratification. Finally, CT with intravenous contrast can provide accurate evaluation of the entire aorta, particularly when performed in an ECG-gated manner. CT is ideally performed prior to conception for assessment of risk in patients with known or suspected aortic disease. If deemed necessary during pregnancy, prospective ECG-gating can dramatically reduce the amount of radiation delivered to the patient and fetus.

## Author Contributions

HI drafted portions of the manuscript and revised the manuscript. AB drafted portions of the manuscript, revised the manuscript, and prepared images. JL outlined and revised the manuscript. All authors contributed to the article and approved the submitted version.

## Conflict of Interest

The authors declare that the research was conducted in the absence of any commercial or financial relationships that could be construed as a potential conflict of interest.

## Publisher’s Note

All claims expressed in this article are solely those of the authors and do not necessarily represent those of their affiliated organizations, or those of the publisher, the editors and the reviewers. Any product that may be evaluated in this article, or claim that may be made by its manufacturer, is not guaranteed or endorsed by the publisher.
